# 197. Healthcare Resource Utilization and Costs Associated with Inappropriate and Suboptimal Prescribing of Antibiotics for Uncomplicated Urinary Tract Infection in the United States

**DOI:** 10.1093/ofid/ofab466.197

**Published:** 2021-12-04

**Authors:** Rena Moon, Alen Marijam, Fanny S Mitrani-Gold, Daniel C Gibbons, Alex Kartashov, Ning Rosenthal, Ashish V Joshi

**Affiliations:** 1 Premier Applied Sciences, Premier Inc., Charlotte, NC; 2 GlaxoSmithKline plc., Collegeville, PA; 3 GlaxoSmithKline plc, Collegeville, PA; 4 GlaxoSmithKline plc., Brentford, Middlesex, UK

## Abstract

**Background:**

Despite well-established guidelines for urinary tract infection (UTI) treatment, prescribing practices vary. We examined the association between inappropriate (IA) or suboptimal (SO) antibiotic (AB) prescribing (RX) and hospitalization, healthcare resource use (HRU), and costs among patients with uncomplicated UTI (uUTI) in the US.

**Methods:**

This retrospective cohort study used linked Premier Healthcare/Optum claims data from female outpatients (≥ 12 years old) with a uUTI diagnosis (January 1, 2013 to December 31, 2018). Patients with complicated UTIs (eg, urological abnormalities, medications/procedures associated with complicated UTI, or intravenous AB receipt at index) were excluded. HRU and costs between patients with IA/SO and appropriate and optimal (AP&OP) AB RX (defined in **Table 1**) were assessed from Optum claims data during index episode (within 28 days of index) and 12-month follow-up.

Table 1. Definitions of appropriateness of AB RX

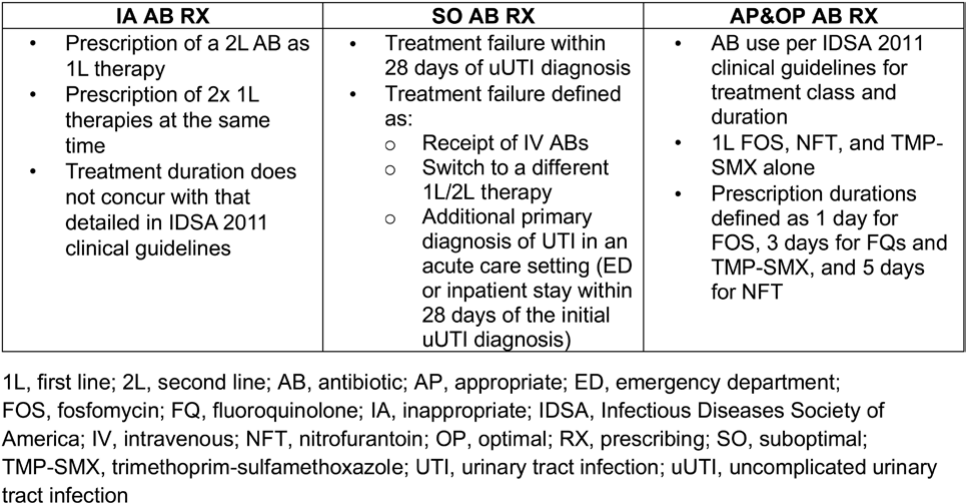

**Results:**

Of 5870 patients, 1856 (31.6%) had IA and 1255 (21.4%) had SO AB RX. Patients with IA/SO AB RX (47.1%) were older and more likely to have a Charlson Comorbidity Index score > 0 than those with AP&OP AB RX (52.9%; **Table 2**). During index episode, mean ambulatory care and pharmacy claims were significantly higher for IA/SO versus AP&OP AB RX (8.0 vs 6.3, 3.3 vs 2.6, respectively; p < 0.01), and total HRU cost per patient was higher for IA/SO (&2616) versus AP&OP AB RX (&649; p < 0.01). During follow-up, 267 (9.7%) patients with IA/SO AB RX had a UTI-related emergency department (ED) visit versus 202 (6.5%) patients with AP&OP AB RX (p < 0.001). Mean UTI-related HRU costs were significantly higher for IA/SO (&5048) versus AP&OP AB RX (&3633; p = 0.01). After adjusting for patient characteristics, patients with IA/SO AB RX were 40% more likely than those with AP&OP AB RX to have a UTI-related ED visit (odds ratio 1.40; 95% confidence interval 1.15–1.71) during follow-up (**Table 3**). Adjusted HRU costs for IA/SO AB RX (vs AP&OP) were numerically higher for index uUTI episode (by &1772), and UTI-related (by &1102) and all-cause (by &1528) charges during follow-up (**Figure**).

Table 2. Baseline characteristics of patients stratified by appropriateness of AB RX

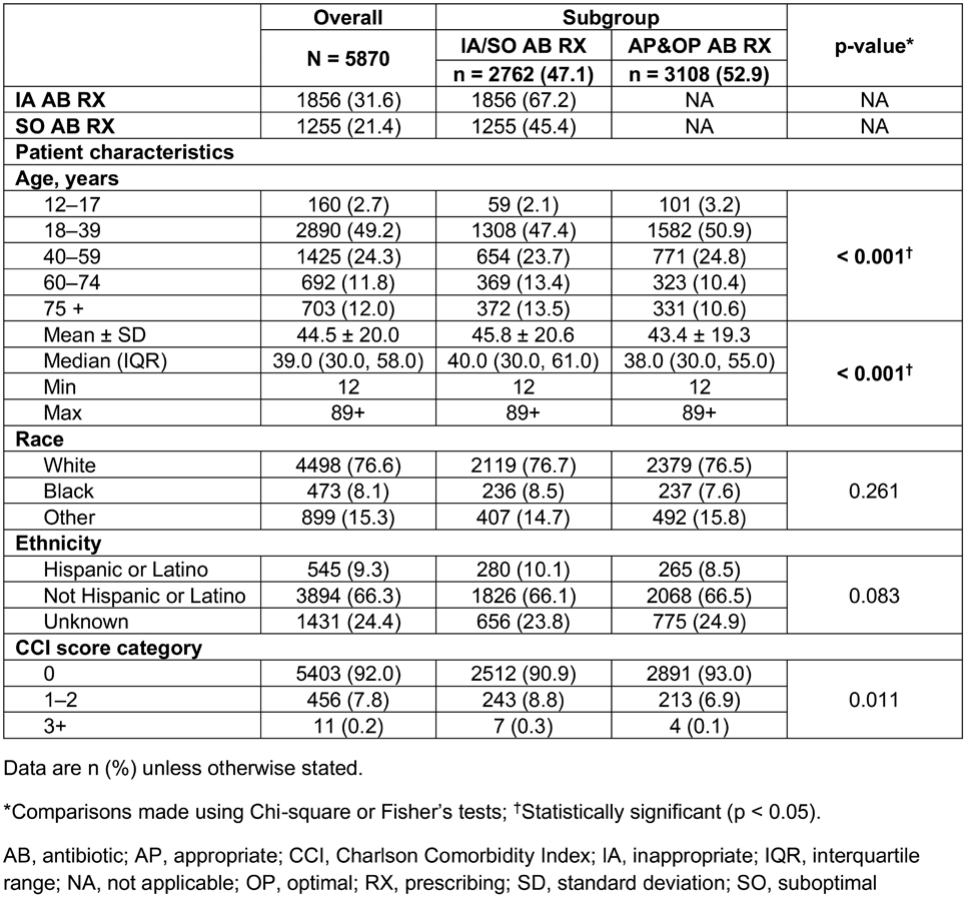

Table 3. Associations of AP&OP AB RX and HRU charges for index episode and 12-month follow-up

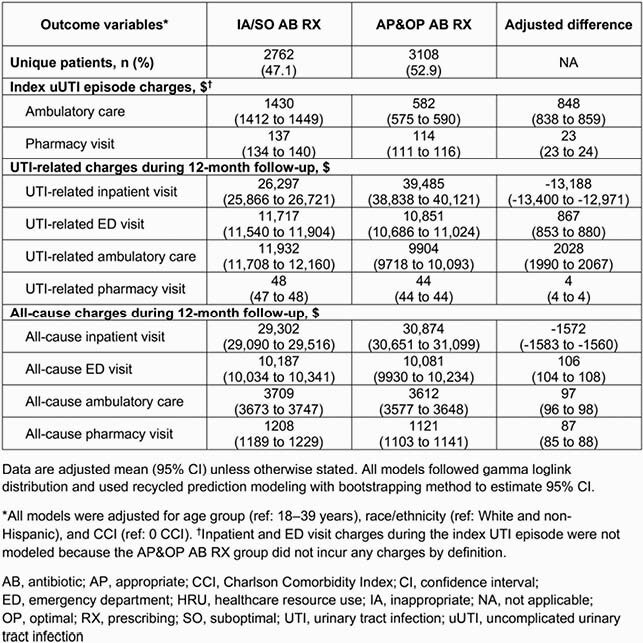

Figure. Total 12-month UTI-related and all-cause visit charges (adjusted), stratified by appropriateness of AB RX at index and during follow-up

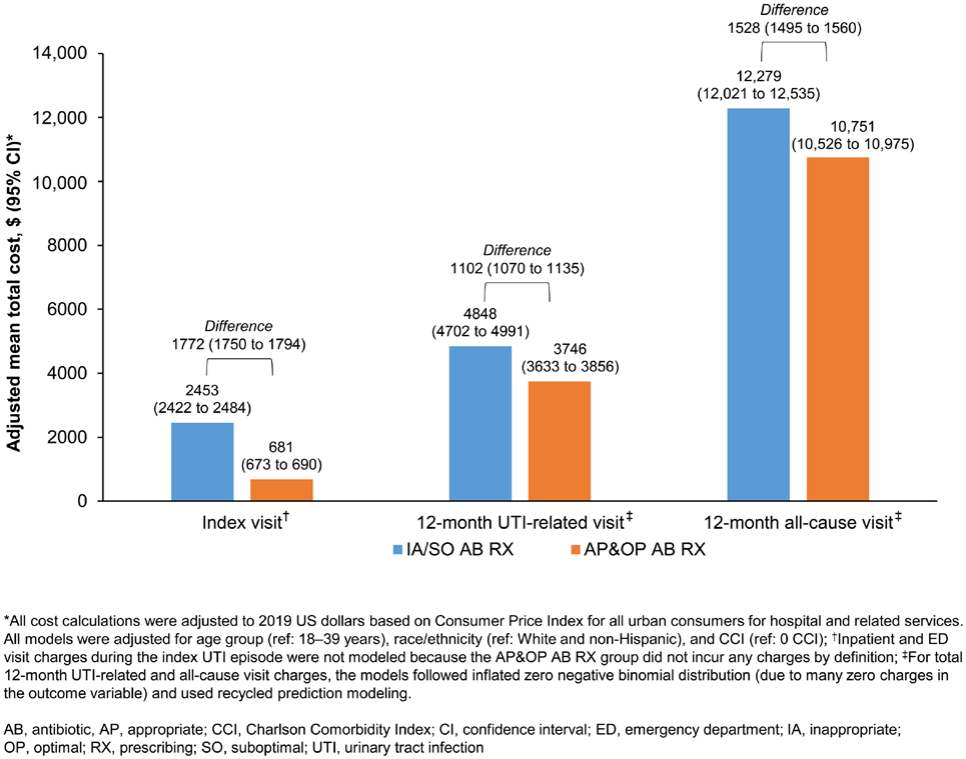

**Conclusion:**

IA/SO AB RX was associated with higher overall and UTI-related HRU and costs during index episode and 12-month follow-up, highlighting a need for education on applying prescription guidelines and the use of culture-based RX.

**Disclosures:**

**Rena Moon, MD**, **Premier Applied Sciences, Premier Inc.** (Employee) **Alen Marijam, MSc**, **GlaxoSmithKline plc.** (Employee, Shareholder) **Fanny S. Mitrani-Gold, MPH**, **GlaxoSmithKline plc.** (Employee, Shareholder) **Daniel C. Gibbons, PhD**, **GlaxoSmithKline plc.** (Employee, Shareholder) **Alex Kartashov, PhD**, **Premier Applied Sciences, Premier Inc.** (Employee) **Ning Rosenthal, MD**, **Premier Applied Sciences, Premier Inc.** (Employee, Shareholder) **Ashish V. Joshi, PhD**, **GlaxoSmithKline plc.** (Employee, Shareholder)

